# Animal production under climate change: a global scientometric analysis of research structure, thematic evolution, and knowledge gaps

**DOI:** 10.1007/s11250-026-05071-0

**Published:** 2026-05-28

**Authors:** Robson Mateus Freitas Silveira, Concepta McManus, Iran José Oliveira da Silva

**Affiliations:** 1 Environment Livestock Research Group (NUPEA), Department of Biosystems Engineering, “Luiz de Queiroz” Agriculture College (ESALQ), University of São Paulo (USP), Piracicaba, SP 13418-900 Brazil; 2 Center for Nuclear Energy in Agriculture (CENA), University of São Paulo (USP), Av. Centenário, 303 - São Dimas, Piracicaba, SP 13416-000 Brazil

**Keywords:** Sustainability, Heat stress, Greenhouse gases, Adaptation, Food security, Animal welfare

## Abstract

Climate change is a major driver of transformation in livestock systems; however, existing reviews remain fragmented, often addressing environmental impacts or adaptation strategies in isolation, without systematically integrating the structure, evolution, and knowledge gaps of the field. This study addresses this limitation through a comprehensive bibliometric–scientometric analysis of global research on climate change and animal production. A total of 1,694 peer-reviewed articles and reviews indexed in Scopus (1974–2025) were retrieved using a structured search applied to titles, abstracts, and keywords. Data were processed through duplicate removal and keyword harmonization, and analyzed using Bibliometrix (R) and VOSviewer to perform co-occurrence network analysis, thematic clustering, and temporal trend evaluation. Results indicate a sustained annual growth rate of 9.47% and increasing international collaboration (35.71%), reflecting the rapid expansion of the field. The co-occurrence network reveals a highly interconnected structure, with “climate change” acting as the central organizing concept linking environmental, physiological, genetic, and production-related domains. Thematic analysis shows that research on greenhouse gas emissions and environmental impacts is well established, whereas emerging areas—such as climate-smart agriculture, One Health, and integrated sustainability frameworks—remain less connected to applied and policy-oriented research. Temporal trends highlight a shift, particularly after 2015, from impact-oriented studies toward more integrated approaches incorporating sustainability, animal welfare, resilience, and adaptive management, alongside increasing use of digital tools such as modeling and machine learning. In addition, life cycle modeling further indicates that the field remains in an early expansion stage, having reached approximately 11.6% of its estimated saturation level, with continued growth expected over the coming decades. Despite this progress, important gaps persist, particularly regarding the translation of scientific knowledge into practice and the uneven geographic distribution of research efforts. Strengthening region-specific and socially inclusive research, enhancing the integration between technological innovation and field-level application, and advancing interdisciplinary frameworks are key priorities to improve the adaptive capacity of livestock systems. By mapping the structure, evolution, and gaps of the field, this study provides a robust basis to inform future research agendas and support the transition toward more resilient and sustainable livestock systems under climate change.

**Graphic abstract:**

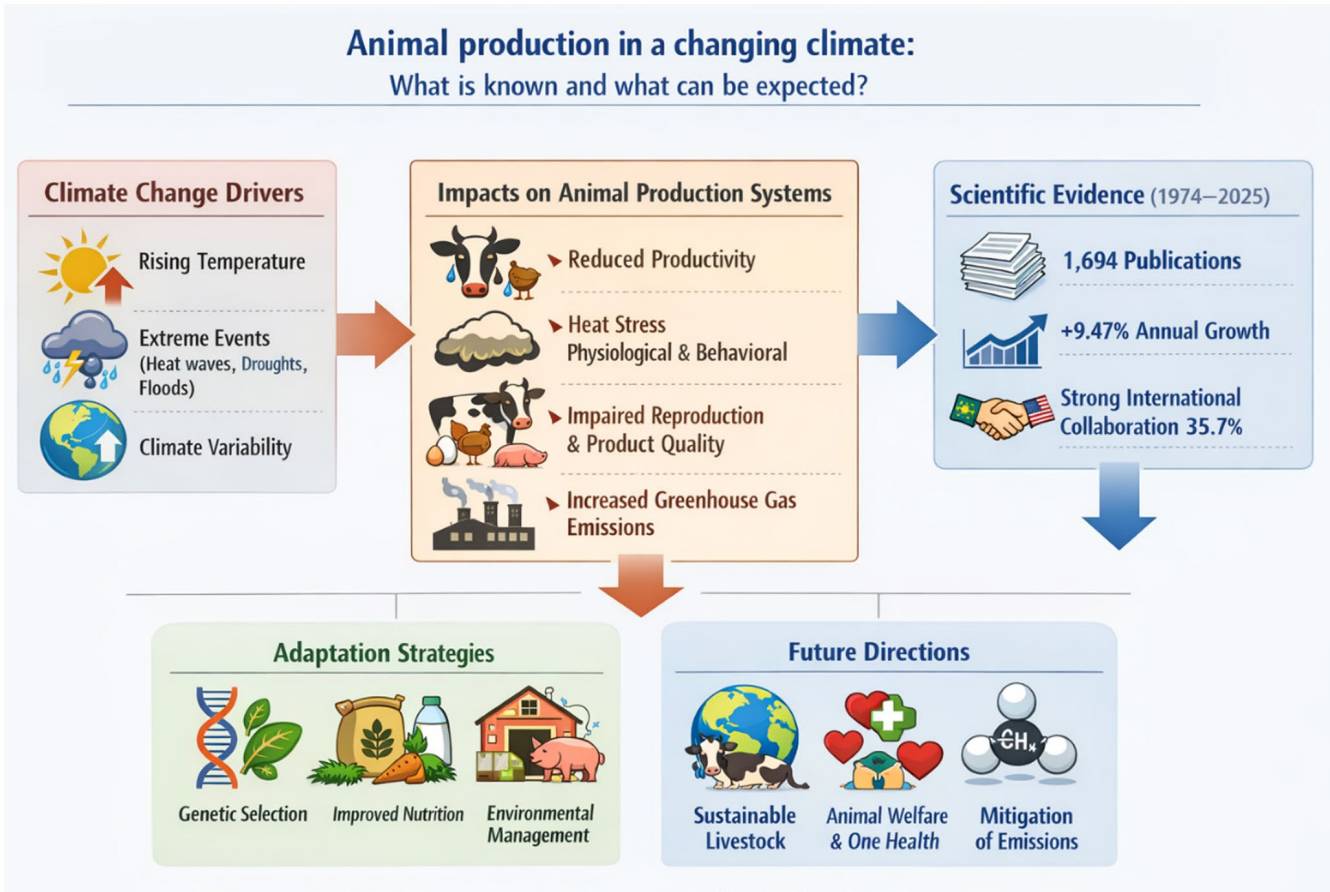

## Introduction

Animal production faces one of its greatest contemporary challenges: adapting to the rapid and profound climate changes underway. Rising average temperatures, the intensification of extreme events (heat waves, droughts, and floods), and greater variability in water regimes directly impact livestock performance, animal welfare, the availability of natural resources, and the stability of production chains (Herrero et al. 2016; Rojas-Downing et al. 2017; Silveira et al. 2026). These factors make it urgent to understand how climate affects livestock systems and what adaptive responses can be adopted, establishing this area as a scientific, political, and productive priority on a global scale (Gerber et al. 2013; Cheng et al. 2022).

In recent decades, the scientific literature on climate change and animal production has grown significantly, addressing issues ranging from the impacts of heat stress on animal metabolism, physiology, and behavior to its effects on reproduction, product quality, and greenhouse gas emissions (Gonzalez-Rivas et al. 2020; Bilal et al. 2021; Herrero et al. 2016; Zenda 2025). Adaptation strategies, such as genetic selection for hardiness, formulating more efficient diets, and improving the animal’s environment, have been highlighted as alternatives to reduce vulnerabilities (Prates 2025). However, the complexity of the topic requires a multidisciplinary and integrated approach, as the responses of production systems vary according to species, production system, and regional context (Onagbesan OM et al. 2023; Silveira et al. 2025a).

Despite the growing body of literature on climate change and animal production, important limitations persist that hinder the advancement of the field. Current knowledge remains fragmented across disciplines, with studies often focusing on isolated components—such as physiology, nutrition, or environmental management—without integrating these dimensions into a systemic understanding of livestock responses to climate change (Herrero et al. 2016; Cheng et al. 2022). Another critical gap lies in the lack of synthesis regarding how research themes have evolved over time, how different domains of knowledge are interconnected, and which areas remain underexplored. Although numerous studies address specific impacts of heat stress or propose adaptation strategies (Sejian et al. 2018; Silveira et al. 2026; Lima et al. 2022), there is still no comprehensive mapping of the intellectual structure of this research field, nor a clear identification of emerging trends, thematic clusters, and knowledge gaps that could guide future investigations.

In this context, a bibliometric approach becomes particularly relevant, as it allows for the systematic evaluation of large volumes of scientific data, enabling the identification of publication patterns, collaboration networks, thematic evolution, and research fronts. Unlike traditional narrative reviews, bibliometric analyses provide quantitative and reproducible insights into the structure and dynamics of a research field, which is especially important in rapidly expanding and multidisciplinary domains such as climate change and animal production (Vieira and McManus 2023; McManus et al. 2023). Moreover, given the accelerated growth of publications in recent years and the increasing complexity of the topic, such an approach is essential to consolidate existing knowledge, identify strategic gaps, and support evidence-based decision-making in both research and policy contexts.

This study aims to analyze, through a bibliometric approach, the intellectual structure, temporal evolution, and main thematic axes of scientific production on climate change and animal production, identifying publication patterns, collaboration networks, research clusters, and knowledge gaps, in order to provide guidance for future investigations and support the development of more effective adaptation and mitigation strategies in the livestock sector.

## Materials and methods

### Overview

To map the scientific landscape on the impacts of climate change on livestock production, we performed a bibliometric and scientometric analysis focused on the co-occurrence of keywords in peer-reviewed literature. This approach was selected due to its ability to systematically synthesize large volumes of scientific information, allowing the identification of knowledge structures, emerging themes, and conceptual relationships in complex and interdisciplinary fields. In particular, keyword co-occurrence analysis enables the detection of latent semantic patterns and the organization of research domains based on the frequency and strength of term associations. The methodology involved structured searches in international databases, preprocessing of bibliographic data, and subsequent visualization using network-based clustering techniques.

### Data collection

The bibliographic data were retrieved from the Scopus^®^ database, recognized for its comprehensive coverage of peer-reviewed scientific publications. Scopus was selected due to its broad multidisciplinary scope, standardized metadata, and compatibility with bibliometric tools such as VOSviewer, which enhances reproducibility and analytical consistency.

The search strategy was designed to capture studies addressing climate-related challenges in livestock systems, using the following query applied to titles, abstracts, and keywords (Table 1).

**Table 1:** Search strategy used in the scopus database

Component	Description
Database	Scopus
Search field	TITLE-ABS-KEY
Core search string	(“climate change” OR “global warming”) AND (“livestock production” OR “animal production” OR “animal agriculture”) AND (“impact” OR “effects” OR “challenges” OR “projections” OR “future scenarios”)
Subject areas	Agricultural and Biological Sciences (AGRI); Environmental Science (ENVI); Social Sciences (SOCI); Veterinary (VETE); Earth and Planetary Sciences (EART); Engineering (ENGI); Biochemistry, Genetics and Molecular Biology (BIOC); Economics, Econometrics and Finance (ECON); Business, Management and Accounting (BUSI); Multidisciplinary (MULT); Computer Science (COMP); Immunology and Microbiology (IMMU); Chemical Engineering (CENG); Physics and Astronomy (PHYS)
Document types	Article (ar); Review (re)
Language	English

Scopus was selected as the primary database due to its broad multidisciplinary coverage, standardized indexing system, and extensive citation metadata, which make it particularly suitable for bibliometric and scientometric analyses. Compared to other databases, Scopus provides more consistent metadata structures and wider journal coverage in agricultural and environmental sciences, supporting robust network and trend analyses.

Metadata, including titles, authors, keywords, affiliations, abstracts, and citation information, were exported in CSV format for further analysis. Data processing and analysis were conducted using the R software with the *bibliometrix* package (Aria and Cuccurullo 2017), complemented by VOSviewer for network visualization and clustering (Van Eck and Waltman 2010).

Prior to analysis, a data cleaning procedure was performed to ensure consistency and reliability. This included the removal of duplicate records, standardization of author names and institutional affiliations, and harmonization of keywords using a thesaurus file.

To ensure data consistency, records from 2025 were treated as a partial year, and analyses involving temporal trends were interpreted with caution to avoid overrepresentation biases associated with incomplete annual data.

It is important to acknowledge that the exclusive use of the Scopus database and English-language publications may introduce selection and linguistic biases, potentially underrepresenting relevant studies published in other languages or indexed in alternative databases such as Web of Science or regional repositories. Furthermore, although Scopus offers extensive coverage, it may still omit locally relevant or non-indexed research, particularly from developing regions, which reinforces the need for cautious interpretation of global patterns.

### Keyword co-occurrence and network visualization

To explore the semantic structure of the research field, a co-occurrence analysis of author keywords and indexed keywords was conducted. This analysis identifies the most frequently occurring terms and their co-appearance within the same documents, which reflects thematic proximity.

Prior to analysis, a data preprocessing step was conducted, including the removal of duplicate records, standardization and harmonization of keywords (e.g., merging synonyms, unifying singular and plural forms, and correcting spelling variations), and the exclusion of non-informative or overly generic terms. Additionally, thresholds were applied to include only keywords with a minimum frequency of occurrence (5 occurrences), ensuring robustness and interpretability of the network.

The resulting keyword network was visualized using VOSviewer^®^, a specialized software for constructing and analyzing bibliometric maps. The clustering of keywords was performed using the clustering algorithm implemented in VOSviewer, based on modularity optimization, with default parameters, allowing the identification of cohesive thematic groups within the network structure.

In this science map:

1. Nodes (colored circles) represent keywords.
2. Edges (connecting lines) indicate the frequency of co-occurrence between terms.
3. Node size corresponds to the individual frequency of each term.

Colors distinguish semantic clusters generated by the clustering algorithm implemented in VOSviewer, representing distinct thematic communities.

### Temporal and thematic trends

Additionally, a temporal trend analysis was performed to examine how the main terms evolved over time. In this visualization, each horizontal blue bar represents the period during which a given term appears in the dataset, while the blue dot marks the central year of occurrence for that term, with its size proportional to its relative frequency.

To enhance interpretability, temporal analyses were conducted considering the normalized frequency of terms across years, minimizing distortions caused by variations in annual publication volume. This representation allows the identification of early and late emerging topics, as well as shifts in the temporal focus of the research field.

### Scientific production life cycle analysis

A temporal growth analysis of scientific production was performed using the *bibliometrix* package in R, aiming to model the evolution and maturity stage of the research field. The cumulative number of publications over time was fitted to a nonlinear sigmoidal growth model, which allows the estimation of key parameters describing the dynamics of knowledge expansion.

The model estimates the theoretical saturation level of publications (K), the inflection point corresponding to the peak growth year (Tₘ), the maximum annual growth rate, and the overall growth duration (Δt). These parameters provide a quantitative framework to characterize the life cycle of the research field, distinguishing phases such as emergence, rapid growth, and maturity.

Model performance was evaluated using goodness-of-fit and information criteria, including the coefficient of determination (R²), root mean square error (RMSE), Akaike Information Criterion (AIC), and Bayesian Information Criterion (BIC). Additionally, milestone years corresponding to different saturation thresholds (10%, 50%, 90%, and 99% of K) were calculated to identify key transition points in the evolution of the field.

Forecasting of future publication trends was conducted based on the fitted model, allowing the projection of cumulative and annual scientific output over time.

## Results

**Fig. 1:**
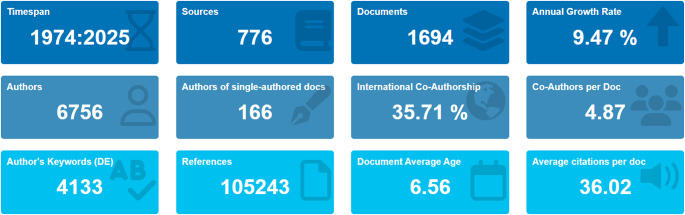
Overview of key indicators from studies evaluating the impact of climate change on animal production

Figure 1 presents a detailed bibliometric overview of scientific production related to the intersection between climate change and animal production, summarizing the main quantitative statistics on the scientific literature published during the analyzed period. In summary, the data demonstrate that scientific production on climate change and animal production has grown consistently and significantly, characterized by strong collaboration between authors and institutions, high interdisciplinarity, great thematic diversity, and considerable impact on academia. This is a structurally consolidated yet still rapidly expanding field, with a growing prominence on global scientific agendas.

The covered period ranged from 1974 to 2025, covering more than five decades of scientific production. During this period, 1,694 articles were identified, published in a total of 776 different sources. The average annual growth rate of scientific production on this topic is 9.47%, indicating a significant and steady increase in interest and relevance in this area of study over time, signaling that the scientific community is responding to the challenges posed by climate change in animal production with a growing volume of publications.

The total number of authors involved in these publications is 6,756, demonstrating a high degree of participation from the international scientific community. Of these, 166 authors published single-authored documents, representing a minority, signaling a predominance of collaborative work in the field. The average co-authorship rate per document is 4.87, reinforcing the collective nature of research developed in this field, which often requires interdisciplinary approaches and cooperation between experts from different fields. Additionally, 35.71% of the publications resulted from collaborations, highlighting the global nature of the issues addressed and the joint efforts of scientists from different countries to address the challenges of animal production under climate change scenarios.

Regarding the content and impact of the publications, the total number of keywords provided by the authors is 4,133, demonstrating the thematic diversity and complexity of the topics covered. The publications cite a total of 105,243 bibliographic references, demonstrating the depth and theoretical foundation of the research. The average dating of the cited documents is 6.56 years, indicating that the publications tend to rely heavily on recent and up-to-date literature, which is common in rapidly evolving scientific fields such as the impact of climate change. The average number of citations per document was 36.02, indicating a relatively high citation performance when interpreted in the context of bibliometric studies in animal science and related fields, where citation impact is inherently field- and time-dependent. This average reveals that the work produced in this area has generated interest and is widely used as reference by other researchers, reinforcing the academic relevance of the topic analyzed.

**Fig. 2:**
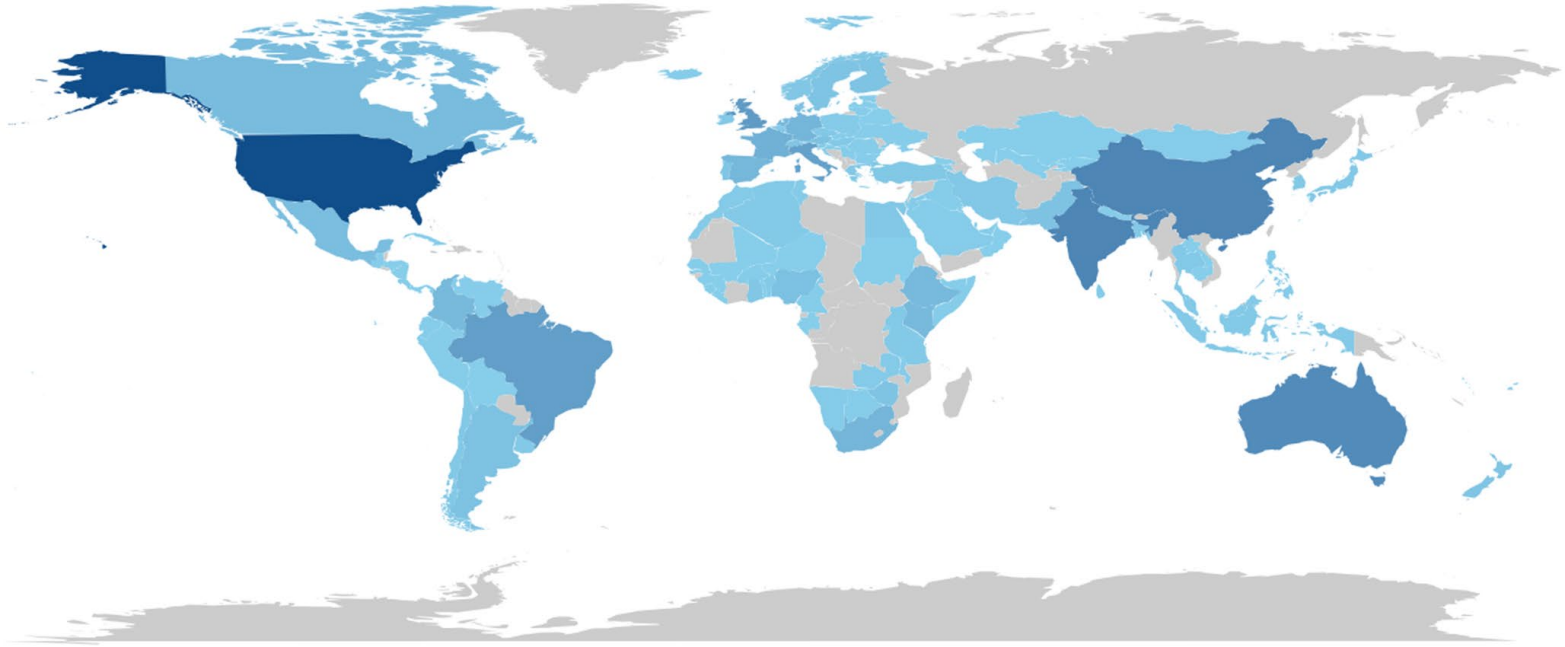
Geographic distribution of global scientific production assessing the impact of climate change on animal production

Figure 2 presents a thematic world map illustrating the geographic distribution of scientific production studying the impact of climate change on animal production. Each country is colored with different intensities of blue, indicating its relative contribution in terms of number of publications. The darker the shade of blue, the greater the volume of scientific production associated with the country. Countries displayed in gray do not necessarily indicate a complete absence of research activity, but may reflect limitations related to database coverage, indexing practices, or the inclusion criteria adopted (e.g., language and source restrictions), which can lead to the underrepresentation of certain regions. Overall, an uneven geographic distribution of scientific production is observed, with a concentration in countries that combine strong research infrastructure, higher investment in science, and strategic interest in agricultural and environmental issues.

There is a strong concentration of scientific production in high-income countries and in large emerging economies with consolidated or rapidly expanding research systems. The United States stands out as the leading global scientific producer on this topic, represented by the darkest shade of blue on the map. This prominence is likely associated with high investment in research, extensive scientific infrastructure, and a strong strategic interest in climate change and its impacts on the agricultural sector. In Asia, China and India also stand out with intense colors, reflecting their growing scientific leadership and the central role of these countries in global agricultural production. China’s strong presence can be interpreted in light of the large scale of its production systems, increasing concerns regarding food security under climate change, and sustained governmental investment in strategic research. India, in turn, also demonstrates significant scientific activity, which may be related to the high vulnerability of its agricultural systems and rural populations to climate variability.

In Europe, a strong scientific production is observed in Western countries such as the United Kingdom, France, Germany, Italy, and Spain, all represented by medium to dark shades of blue. These countries have a consolidated tradition in environmental and agricultural research and are actively engaged in international research networks and policy frameworks addressing climate change mitigation and adaptation.

In the Southern Hemisphere, Oceania, specifically Australia, is proving to be an important hub, which may be associated with its exposure to climate-related challenges such as prolonged droughts and its strong investment in adaptive livestock systems. In Latin America, Brazil ranks as the leading country in scientific production, occupying a prominent regional position. The country combines significant biodiversity, extensive agricultural production, and a history of investment in agricultural-environmental research, especially by institutions such as Embrapa and public universities. Other Latin American countries such as Mexico, Colombia, and Argentina also have moderate levels of production, reflecting their respective scientific capacities and agricultural priorities.

Regarding Africa, a more modest contribution is observed, with a greater presence from countries in the south and east of the continent, such as South Africa, Kenya, Ethiopia, and Nigeria. Although many African countries are highly vulnerable to climate change, their scientific production capacity appears to be constrained by structural factors such as limited research funding, infrastructure, and human capital, as well as potential underrepresentation in indexed databases.

Finally, countries in the Middle East, Central Asia, and parts of Eastern Europe and Central Africa appear to have little or no contribution, which should be interpreted with caution, as this pattern may reflect a combination of lower publication visibility in indexed journals, language barriers, and disparities in research investment, rather than an actual absence of scientific activity.

**Fig. 3:**
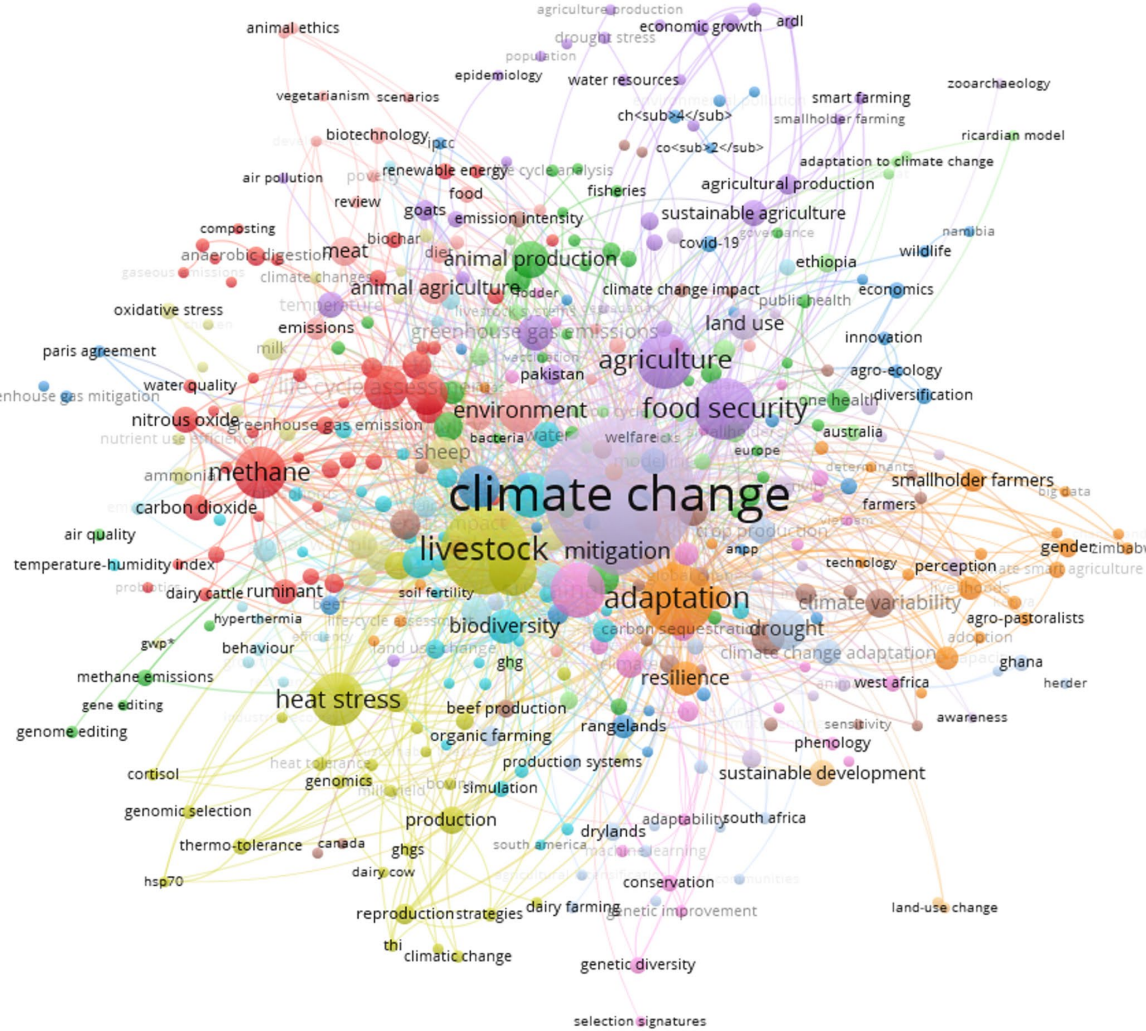
Co-occurrence mapping of terms extracted from scientific publications related to animal production and climate change

Figure 3 presents a co-occurrence analysis of terms extracted from scientific publications related to the impact of climate change on animal production. In summary, this topic is a research field characterized by strong interdisciplinarity, analyzing animal production from multiple perspectives: environmental, physiological, genetic, economic, social, ethical, and technological. Climate change acts as a central axis that articulates all these dimensions, indicating that the challenges of contemporary livestock farming require integrated and transdisciplinary approaches. The focus on adaptation, mitigation, food security, resilience, and technological innovation reveals the direction scientific research is taking in an attempt to build more sustainable agricultural systems prepared for the climate challenges of the present and future.

In the center of the map, the term “climate change” stands out as the most central and widely connected concept, revealing its structuring role in contemporary debates surrounding animal production. Terms such as “livestock”, “adaptation”, “mitigation”, “agriculture”, “food security”, “environment”, and “resilience” appear closely and with significant weight, demonstrating that these themes are strongly associated with discussions about the impacts of climate change and strategies to address them.

Around these central terms, the network is organized into distinct thematic clusters. One of these clusters, represented in red, focuses on greenhouse gases and the environmental impacts of livestock farming. It includes terms such as “methane”, “carbon dioxide”, “nitrous oxide”, “greenhouse gases”, “emissions”, “composting”, and “anaerobic digestion”. This cluster reflects scientific efforts to quantify and mitigate the environmental impacts of animal production, especially regarding methane emissions from ruminants, manure management, and air and water quality.

Another group, located in the lower left of Fig. 3 and represented in yellow, focuses on physiological and genetic aspects related to heat stress. Terms such as “heat stress”, “genomics”, “genomic selection”, “cortisol”, “hsp70”, “thermotolerance”, and “THI” indicate a line of research focused on identifying genetic markers and physiological responses to heat, highlighting the search for animals better adapted to extreme environmental conditions.

The green group is positioned between the central axes and is spread across the entire network, although it shows some concentration. It includes terms such as “animal production,” “life cycle assessment,” “One Health,” “adaptation to climate change,” “livestock systems,” and “fish.” This set of themes represents efforts aimed at reconfiguring animal production systems, seeking to reconcile productivity with environmental, economic, and social sustainability through more efficient management practices, reduced environmental impacts, and the promotion of integrated health among animals, people, and ecosystems.

The purple group brings together terms related to sustainable agriculture, land use, and food security. It includes the terms “agriculture,” “sustainable agriculture,” “land use,” “innovation,” “diversification,” “climate-smart agriculture,” and “smart agriculture.” This set points to the search for more efficient, resilient, and environmentally integrated agricultural practices, capable of responding to global challenges related to climate change, the availability of natural resources, and the stable and accessible supply of food.

On the right side of the grid, the orange cluster addresses the social and territorial dimensions of adaptation. Terms such as “smallholders,” “adaptation,” “perception,” “adaptation to climate change,” “technology adoption,” “resilience,” “smart agriculture,” and “agropastoral” appear. This cluster highlights the importance of considering sociocultural factors, local perceptions, gender inequalities, and other social markers (such as income, race, and generation) in adaptive capacity. It also shows that the adoption of innovations and technologies depends on specific contexts, vulnerability conditions, and the resources available in each territory.

In addition to these clusters, terms such as “simulation,” “machine learning,” “modeling,” and “big data” appear connecting different regions of the network, indicating the growing use of computational and analytical tools to predict scenarios, integrate diverse databases, and support decision-making. These elements function as bridges between the different themes, allowing the articulation of biophysical, economic, and social information and thus guiding more integrated, evidence-based adaptation and planning strategies.

**Fig. 4:**
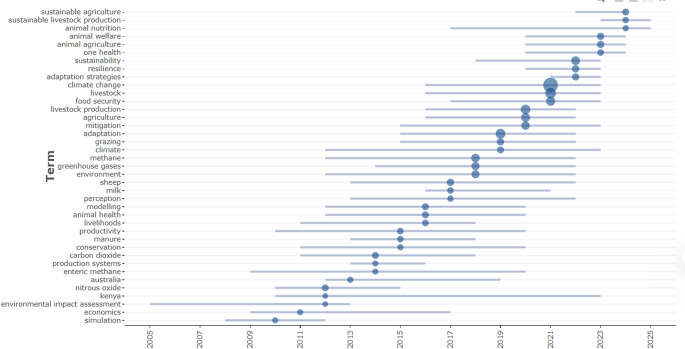
Temporal analysis of the occurrence and evolution of key terms in studies assessing the impact of climate change on animal production

Figure 4 is a temporal analysis of the occurrence and evolution of key terms associated with studies assessing the impact of climate change on animal production. In summary, it highlights the consolidation of an interdisciplinary and dynamic field, in which the themes of sustainability, climate change, resilience, and adaptive strategies have gained centrality in animal production research, especially in the last 10 years. The pattern of emergence and expansion of these terms indicates that future scientific agendas should continue to prioritize the integration of animal science, public health, environment, and sustainable development.

In general, there has been a clear trend of increasing use of terms related to sustainability, climate, and environmental impacts over time, especially after 2010. The most frequently used and visually prominent terms, as indicated by the larger size of the circles, are “climate change”, “sustainability”, “resilience”, “adaptation strategies”, “greenhouse gases”, and “livestock”. These terms gained significant relevance, particularly between 2015 and 2023, indicating that the scientific debate has begun to more intensely incorporate environmental and adaptive issues into animal production systems.

At the top of the figure, terms such as “sustainable agriculture” and “sustainable livestock production” appear, with a timeframe extending to 2025, suggesting that these concepts are the most recently consolidated and likely to remain prominent in the coming years. The presence of these terms demonstrates the scientific community’s growing interest in integrating agricultural practices with environmental, social, and economic sustainability criteria.

Classic terms such as “animal nutrition”, “animal welfare”, and “animal agriculture” have been around for a long time, but their increase intensified since 2015, coinciding with the rise of the term “one health”, which also gained prominence. This term represents an integrated approach to human, animal, and environmental health, and its emergence reinforces the trend toward interconnectivity between different fields of knowledge.

Since 2015, progressive expansion has also been observed in operational terms such as “adaptation strategies”, “mitigation”, “methane”, “greenhouse gases”, “climate”, and “grazing”, demonstrating the increase in studies focused on the role of livestock systems in climate change and strategies to address its effects. These terms are directly related to greenhouse gas emissions from livestock farming and the management practices that can minimize their impacts.

Other relevant terms, such as “food security”, “livelihoods”, “perception”, and “modeling”, reflect more recent approaches integrated with the socioeconomic context and predictive scenario modeling. This analysis reveals that animal production is now being approached not only from a technical-production perspective, but also considering the social effects and the perceptions of those involved in the production chains.

Keywords such as “environmental impact assessment”, “economics”, and “simulation” appear sporadically but represent fundamental approaches for the holistic assessment of production systems and for projecting future scenarios. The presence of these terms since 2005, albeit less frequently, demonstrates the role of quantitative and economic tools in supporting strategic decisions in animal production.

**Fig. 5:**
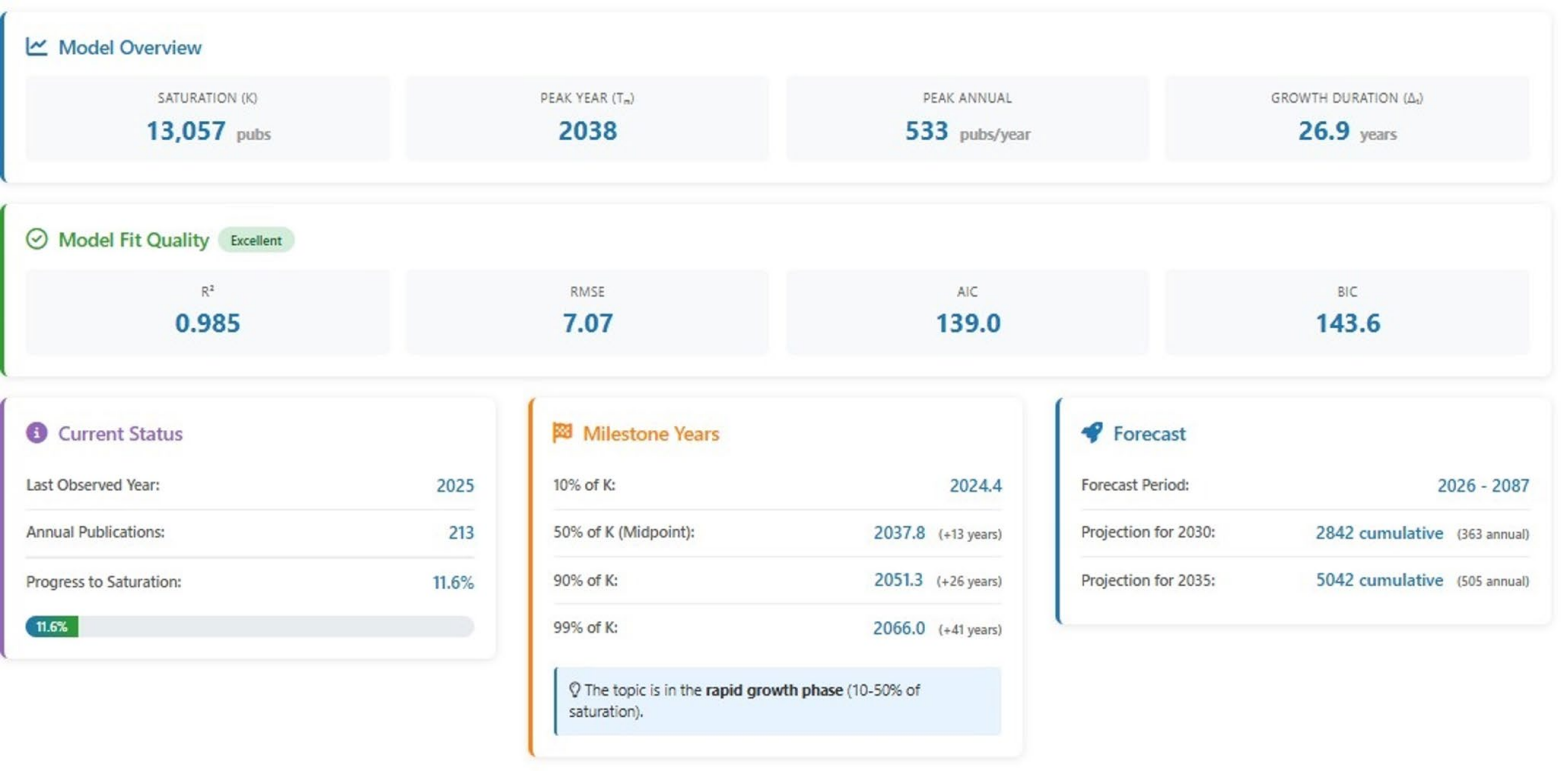
Logistic growth model and projection of scientific production related to climate change and animal production

Figure 5 presents the modeling results of the temporal evolution of scientific production on climate change and animal production. The model indicates a projected saturation level (K) of 13,057 publications, suggesting a substantial future expansion of the field before reaching maturity. The estimated peak year (Tₘ) occurs around 2038, with a maximum annual growth rate of approximately 533 publications per year, reflecting a phase of accelerated knowledge production. The overall growth duration (Δt) is estimated at 26.9 years, indicating a relatively prolonged expansion period characteristic of complex and interdisciplinary research domains.

The model fit quality was classified as excellent (R² = 0.985), demonstrating a high level of agreement between observed and predicted values. Error metrics (RMSE = 7.07) and information criteria (AIC = 139.0; BIC = 143.6) further confirm the robustness and reliability of the model. As of the last observed year (2025), the field had reached only 11.6% of its estimated saturation level, indicating that it remains in an early and rapidly expanding stage of development.

Milestone analysis reveals that the field reached 10% of its projected capacity around 2024, while the midpoint (50% of K) is expected to occur near 2037–2038, marking the transition to a mature phase. Higher saturation thresholds, such as 90% and 99%, are projected for 2051 and 2066, respectively, suggesting long-term consolidation. Forecast scenarios indicate a continued increase in cumulative publications, reaching approximately 2,842 documents by 2030 and 5,042 by 2035. Overall, these results demonstrate that the scientific domain of climate change and animal production is currently in a rapid growth stage (10–50% of saturation), with strong expansion dynamics expected over the next decades.

## Discussion

The analysis of scientific production reveals that the field of sustainable animal production has expanded substantially between 1974 and 2025, with a total of 1,694 documents published by 6,756 authors, corresponding to an annual growth rate of 9.47%. This upward trend reflects increasing global concern about the impacts of climate change on agricultural systems, as well as the growing demand for sustainable solutions supported by scientific evidence and technological advances (Silveira et al. 2025a).

The average of 36.02 citations per document, combined with a remarkable volume of 105,243 references, indicates a consolidated and widely consulted literature, demonstrating the academic relevance and practical applicability of the studies developed. Furthermore, the average dating of the documents (6.56 years) highlights the recent and dynamic nature of the topic, reflecting the incorporation of innovative technologies such as artificial intelligence and machine learning in the agricultural sector.

Another relevant aspect is the degree of international collaboration, with 35.71% of studies conducted in co-authorship between different countries. This trend highlights the globalization of environmental and production concerns, as well as the formation of inter-institutional networks that pursue common objectives, aligned with the goals of the UN 2030 Agenda for Sustainable Development (Silveira et al. 2025b; Horváthné and Zörög 2025).

The diversity of 776 distinct sources and 4133 different keywords reinforces the multidisciplinary and transversal nature of this field, which integrates areas such as animal science, engineering, genetics, environment, economics and public health. This finding is consistent with the bibliometric review conducted by Silveira et al. (2025a).

The analysis of the geographic distribution of scientific publications reveals an asymmetrical pattern in the development of knowledge on sustainable animal production, with a strong concentration in countries of the Northern Hemisphere, particularly the United States, China, and Western European nations. The United States significantly leads the number of scientific articles, followed by China, Brazil, and Australia, reflecting the direct correlation between technological infrastructure, agricultural research funding, and cutting-edge scientific production. This trend is in line with the findings of Silveira et al. (2025a), who emphasized that China has rapidly expanded the number of studies on sustainable animal production, positioning itself as a potential global leader in the coming decades, especially due to the incorporation of artificial intelligence and digital technologies in agriculture. Furthermore, the leadership of developed countries is also related to the ability to establish international collaborative networks, facilitating the circulation and impact of knowledge.

On the other hand, there is a smaller, but still significant, representation of countries in Africa, Central America, parts of Asia, and Eastern Europe—regions that face high vulnerability to the impacts of climate change on agriculture (Zenda 2025). This geographic imbalance goes beyond disparities in publication counts and reflects structural inequalities in global knowledge systems, where peripheral regions are often underrepresented in agenda-setting processes and knowledge validation mechanisms. Such asymmetry reinforces a cycle in which regions most affected by climate change have limited participation in the production of context-specific scientific evidence, thereby constraining the development of locally adapted and scalable solutions. This critical gap in scientific production can be attributed to multiple factors, including limited research investment, restricted access to advanced technologies, difficulties in establishing international collaborations, and reduced visibility in high-impact journals. Consequently, the concentration of scientific leadership in a few countries may lead to a misalignment between global research priorities and local adaptation needs, potentially undermining the effectiveness, equity, and long-term sustainability of mitigation and adaptation strategies in vulnerable regions (Pasgaard and Strange 2013).

This geographic imbalance compromises equality in the generation and dissemination of technological and sustainable solutions, with direct implications for the formulation of public policies and the effectiveness of climate adaptation strategies in livestock systems. In regions with limited scientific production, decision-making processes may rely on generalized or externally derived evidence, which may not adequately reflect local socio-environmental conditions, thereby reducing the efficiency and applicability of proposed interventions, highlighting the need to strengthen local research capacities. Promoting international cooperation, especially those that value knowledge exchange and the creation of contextualized technologies, is essential to expanding scientific inclusion. In this context, strengthening locally grounded research is critical to support evidence-based policies, improve technology transfer, and ensure that adaptation strategies are tailored to the specific vulnerabilities and production systems of each region. Furthermore, incorporating traditional knowledge and local practices is essential for the development of more sustainable and resilient production systems, where the dialogue between modern science and traditional knowledge enhances adaptation to specific socio-environmental conditions (Musara et al. 2021). Such integration can also contribute to more inclusive governance frameworks, enhancing stakeholder engagement and increasing the likelihood of successful implementation of climate-smart livestock practices.

The keyword co-occurrence map reveals the complexity and multidimensionality of the scientific field connecting animal production, climate change, and sustainability. The central term “climate change” reinforces its role as a structuring thematic axis of the entire research network, emphasizing the global importance of the topic for agriculture (Rojas-Downing et al. 2017; Contreras et al. 2025).

Around this core, keywords such as “livestock”, “adaptation”, “food security”, “agriculture”, “environment”, and “heat stress” form interconnected thematic clusters, reflecting the central concerns of sustainable livestock production in the context of climate change. The presence of terms related to greenhouse gases, such as “methane”, “carbon dioxide”, “nitrous oxide”, and “greenhouse gases”, highlights the emphasis on mitigating the environmental impacts of livestock farming, especially reducing emissions, a topic widely discussed in recent studies (Gerber et al. 2013; Herrero et al. 2016; Roques et al. 2024).

The emphasis given to “adaptation”, and “resilience” indicates progress in recognizing the need to prepare production systems to respond to climate variations, since these concepts explicitly address the capacity of animals and production systems to maintain performance under environmental stress. In contrast to earlier approaches focused mainly on quantifying impacts or mitigating emissions, the growing use of these terms reflects a shift toward anticipating climate extremes, enhancing the ability of animals to cope with heat and resource scarcity, and designing management strategies that maintain productivity and welfare across a wider range of climatic conditions (Silveira et al. 2026).

The interconnection between “animal welfare”, “health”, and “sustainable development” signals a growing trend toward the adoption of the ‘One Welfare’ paradigm, in which animal welfare is intrinsically linked to environmental and human health. This integrated approach represents a transition from a purely productivism model to a more holistic and ethical vision of agricultural production, aligned with the UN Sustainable Development Goals (Silveira et al. 2023, 2025a; Mellor 2016).

Furthermore, the presence of terms such as “genomics”, “genetic selection”, “selection signatures”, and “genetic diversity” reveals the strengthening and genetic improvement focused on climate resilience. These emerging technologies enable the identification of genetic traits associated with adaptation and resistance to heat stress, reinforcing the need for integrated strategies involving technology, genetics, and sustainable management to address the challenges posed by climate change (Salvian et al. 2023; Carrara et al. 2023).

However, thematic areas with lower connectivity density, such as “smallholder farmers” and “perception”, indicate gaps in addressing the social and cultural dimensions of the topics addressed. This lack highlights the importance of expanding applied research efforts, especially in local contexts and rural communities, where production systems are predominantly small-scale and face specific challenges related to adaptation and sustainability (Hussein et al. 2024; Nyang’Au et al. 2021).

In Fig. 4, an analysis of the thematic evolution of scientific literature on sustainability in livestock farming over the past two decades reveals a trajectory of consolidation and diversification that reflects the conceptual and technological transformations of the field. The sharp growth in publications since 2010, especially on topics related to adaptation, emissions, and resilience, highlights the intensification of scientific interest in understanding and mitigating the impacts of climate change on animal production.

In the first years analyzed, from 2008 to 2012, the literature focused predominantly on mitigating the environmental impacts of livestock farming, with emphasis on terms such as “greenhouse gases”, “climate”, “methane”, and “emissions”. This initial emphasis is aligned with global discussions on global warming and the role of ruminant animals in greenhouse gas emissions, as evidenced by Roques et al. (2024) and Herrero et al. (2016). This focus reflects the urgency of quantifying and reducing emissions to contribute to international climate goals.

Beginning in 2015, a significant thematic shift was observed, with the emergence and strengthening of terms such as “adaptation strategies”, “resilience”, “sustainability”, “animal welfare”, and “one health”. This shift indicates a broadening of the scientific agenda, which now incorporates not only mitigation but also integrated and adaptive responses to climate challenges. Silveira et al. (2023) highlight this trend by proposing the concept of a sustainable animal as one that combines productive performance, environmental adaptation, and health, highlighting the need for multidisciplinary and integrative approaches to address the complex challenges of contemporary agriculture.

In recent years, especially since 2020, the growing presence of terms such as “one health”, “animal ethics”, “smart farming”, “climate-smart agriculture”, and “sustainable livestock production” highlights the adoption of a systemic and interdisciplinary perspective. This approach connects animal welfare, public health, technology, and environmental sustainability, reflecting a “new” vision of agricultural production (Silveira et al. 2025a). The incorporation of digital tools, indicated by the emergence of terms such as “simulation”, “modeling”, and “RNA-seq”, demonstrates the technological advancement and methodological sophistication employed to forecast scenarios and guide adaptive strategies, as pointed out by Rahimi et al. (2021); Astuti et al. (2024) and Silveira et al. (2026).

Furthermore, the consistent, though less frequent, presence of terms related to “environmental impact assessment” and “economics” reveals a growing concern with the socioeconomic viability of proposed solutions. This indicates that sustainability in livestock farming is being understood in an increasingly multidimensional way, integrating environmental, economic, and social aspects, in line with the Sustainable Development Goals (SDGs) and the One Welfare approach, which emphasizes the interdependence between animal, human, and environmental health (Gerber et al. 2013; Silveira et al. 2025a).

The life cycle analysis of scientific production reveals that research on climate change and animal production is still in a dynamic expansion phase, far from reaching its theoretical maturity. The projection that the field has achieved only a small fraction of its saturation level, combined with a peak expected in the coming decades, indicates not only sustained scientific interest but also the increasing complexity and urgency of the topic. This growth trajectory reflects the progressive integration of traditionally fragmented domains, such as physiology, environmental sciences, genetics, and socioeconomics, into more systemic and interdisciplinary research frameworks. At the same time, the extended growth duration suggests that the field will continue to evolve alongside global challenges, particularly those related to climate variability, food security, and sustainability transitions.

## Conclusion

This study provides a comprehensive synthesis of the scientific evolution of research on climate change and animal production, demonstrating a consistent expansion of knowledge characterized by increasing interdisciplinarity, methodological sophistication, and thematic diversification. The results indicate that scientific production has progressively shifted from a predominantly impact-oriented perspective toward more integrated approaches that incorporate adaptation, resilience, animal welfare, and sustainability.

Despite this advancement, the field remains in an active expansion phase rather than a fully mature stage, as indicated by the life cycle analysis of scientific production. This suggests that, although the knowledge base is rapidly growing and becoming more structured, significant opportunities remain for further conceptual consolidation and empirical development.

A critical gap persists between scientific evidence and its practical implementation in livestock systems. While the literature increasingly proposes technological, genetic, and management-based solutions, their adoption remains uneven across regions, particularly in vulnerable areas where structural, economic, and institutional constraints limit the translation of knowledge into practice.

The findings also highlight persistent geographical asymmetries in scientific production, with underrepresentation of regions that are simultaneously the most exposed to climate risks. This imbalance reinforces the need to strengthen research capacity, foster inclusive knowledge systems, and promote equitable participation in global scientific agendas. In this context, socio-environmental justice emerges as a central dimension, as adaptation and mitigation strategies must consider local realities, smallholder systems, and historically marginalized groups.

From a governance perspective, the transition toward sustainable livestock systems depends not only on technological innovation but also on the development of institutional arrangements capable of integrating scientific knowledge with policy design and stakeholder engagement. Strengthening science–policy–practice interfaces, improving data accessibility, and promoting collaborative and context-specific approaches are key elements to enhance the effectiveness of adaptation strategies.

Future research should prioritize the regionalization of studies, the integration of socio-economic and cultural dimensions, and the evaluation of implementation pathways under real-world conditions. Advancing interdisciplinary frameworks that connect animal science, environmental science, and social sciences will be essential to address the complexity of livestock systems under climate change.

In summary, this study shows that the scientific field is dynamically evolving and structurally consolidating, but still far from reaching its theoretical maturity. Its long-term impact will depend on the capacity to bridge the gap between evidence and implementation through inclusive governance, context-sensitive solutions, and equitable development pathways.

## Data Availability

The dataset generated from Scopus is available from the corresponding author upon reasonable request.

## Declarations

### Generative AI and AI-assisted technologies

During the preparation of this manuscript, the authors used ChatGPT to assist with readability improvement, language refinement, and manuscript organization. The manuscript was critically reviewed, revised, and validated by the authors, who assume full responsibility for the accuracy, originality, and integrity of the content. The graphical abstract was created with the assistance of AI-based image generation tools and subsequently reviewed, refined, and validated by the authors to ensure scientific accuracy and consistency with the manuscript content.
